# Multi-layered epigenetic mechanisms contribute to transcriptional memory in T lymphocytes

**DOI:** 10.1186/s12865-015-0089-9

**Published:** 2015-05-06

**Authors:** Jennifer Dunn, Robert McCuaig, Wen Juan Tu, Kristine Hardy, Sudha Rao

**Affiliations:** Faculty of Education, Science, Technology & Maths, University of Canberra, Canberra, ACT Australia

**Keywords:** Transcriptional memory, Memory T cells, Epigenetics, Post-translational modification, Histone variant exchange, PKC-theta, Yeast

## Abstract

**Background:**

Immunological memory is the ability of the immune system to respond more rapidly and effectively to previously encountered pathogens, a key feature of adaptive immunity. The capacity of memory T cells to “remember” previous cellular responses to specific antigens ultimately resides in their unique patterns of gene expression. Following re-exposure to an antigen, previously activated genes are transcribed more rapidly and robustly in memory T cells compared to their naïve counterparts. The ability for cells to remember past transcriptional responses is termed “adaptive transcriptional memory”.

**Results:**

Recent global epigenome studies suggest that epigenetic mechanisms are central to establishing and maintaining transcriptional memory, with elegant studies in model organisms providing tantalizing insights into the epigenetic programs that contribute to adaptive immunity. These epigenetic mechanisms are diverse, and include not only classical acetylation and methylation events, but also exciting and less well-known mechanisms involving histone structure, upstream signalling pathways, and nuclear localisation of genomic regions.

**Conclusions:**

Current global health challenges in areas such as tuberculosis and influenza demand not only more effective and safer vaccines, but also vaccines for a wider range of health priorities, including HIV, cancer, and emerging pathogens such as Ebola. Understanding the multi-layered epigenetic mechanisms that underpin the rapid recall responses of memory T cells following reactivation is a critical component of this development pathway.

## Introduction

Memory T lymphocytes are key cells in the adaptive immune system that are essential for fighting off attacking pathogens. T cells eliminate pathogens by stimulating B cells to produce antibodies (CD4^+^ helper T cells), killing infected host cells (CD8^+^ cytotoxic T cells), secreting cytokines that inhibit pathogen replication, and promoting inflammation. Upon antigenic stimulation, naïve T cells undergo massive clonal expansion to generate large numbers of effector T cells. Following antigen clearance, the majority (90-95%) of effector T cells undergo apoptosis, whilst the remaining pool of activated cells persist to establish a small population of memory T cells [1]. Memory T cells can be divided into two main subsets: central memory T cells (T_CM_ cells) and effector memory T cells (T_EM_ cells), each defined by their cell surface phenotype [2]. T_CM_ cells retain the migratory characteristics of naïve T cells (circulating secondary lymphoid organs) and can differentiate and proliferate after antigenic re-stimulation. In contrast, T_EM_ cells resemble their effector counterparts (circulating to non-lymphoid tissues), and express higher levels of effector molecules. Despite the functional differences between these memory T cell subsets, they both share the common feature of being able to establish immunological memory.

A distinctive feature of memory T cells is that they transcribe distinct cohorts of inducible genes more rapidly and at greater levels than their naïve and effector counterparts, in response to reinfection. These genes include a number of well-described immune-responsive genes, such as interleukin-2 (*IL-2*), tumour necrosis factor (*TNF*), and interferon-γ (*IFNG*). Genes that are highly expressed in memory cells can be further divided into three functional groups: genes involved in immune function (such as T cell activation, migration, intracellular signalling, and effector molecules), genes that promote memory T cell survival and homeostasis (cytokines/chemokines and receptors), and genes encoding transcriptional regulators with multiple and diverse functions [3]. Although most of these genes are shared between different memory T cell subsets, subtype-specific differences exist. For example, CD8^+^ memory T cells express higher levels of genes encoding important effector molecules, such as granzymes and killer cell lectin-like receptors [3]. Similarly, T_EM_ cells express higher levels of certain genes than T_CM_ cells, including those encoding lectins, MHC class II molecules, cell surface receptors, and cytotoxic molecules (e.g., *IFNG*) [3]. The differential expression of these genes explains the unique and differing functions of memory T cells and their subsets.

The rapid and abundant inducible gene expression in memory T cells is termed ‘transcriptional memory’. Remarkably, memory T cells are able to retain transcriptional memory up to 75 years after their first encounter with an antigen [4]. Thus, unravelling the molecular programs that lead to memory T cell generation in response to infections is relevant for vaccine development and understanding T cell-mediated diseases.

The feature of transcriptional memory is not unique to memory T cells, but is evolutionarily conserved in eukaryotic organisms. For example, yeast cells are able to elicit transcriptional memory responses to galactose; following glucose repression, the *GAL1* gene (encoding galactokinase) is transcribed more rapidly in cells previously exposed to galactose compared to galactose-naïve cells [5]. Whilst T cells have been an excellent model system for studying transcriptional memory, lower eukaryotes have also been an excellent resource for untangling the mechanisms that control transcriptional memory, not least because they are amenable to elegant genetic analyses (see below). Although the cellular features of immunological memory are well defined, the epigenetic programs underpinning transcriptional memory are rather less well understood.

In this review, we summarise current knowledge on the contribution of different multi-layered epigenetic mechanisms to eliciting transcriptional memory programs in T cells. We also discuss the roles of variant histones, chromatin chaperone/remodelling proteins, and the emerging class of chromatin-associated kinases in regulating transcription in T memory cells.

### Epigenetic “marks” prime genes for future transcription in memory T cells

Epigenetic regulation is defined as heritable modifications to DNA and histone proteins that modulate gene expression in the absence of base sequence changes [3,6]. These epigenetic changes are crucial for determining and maintaining cell fate during development. The contribution of the post-translational modification (PTM) landscape to memory T cell development has been intensely studied over the past decade. Histone modifications (or PTMs; histone or epigenetic “marks”) are interdependent, switching genes on and off in response to extracellular signals [7]. These marks alter transcriptional activity by changing the chromatin composition to expose or hide target genes from polymerases and other transcriptional machinery [8]. Epigenetic marks recruit histone-modifying enzymes that alter chromatin accessibility, in turn generating numerous chromatin “platforms” that regulate gene expression and the recruitment of protein complexes [7]. Acetylation, methylation, phosphorylation, and ubiquitinylation are common PTMs involved in transcriptional regulation [8,9]. Acetylation of N-terminal histone tails is predominately associated with open chromatin states (e.g., acetylation of H3K9, H3K14, H4K5, and H4K16) conducive to active gene transcription [1]. In contrast, histone methylation is more complex and results in different effects on transcription depending on the extent of methylation (i.e., mono-, di-, or trimethylation). For example, monomethylation of H3K9, H3K27, and H3K79 histone proteins is associated with euchromatin states, whereas trimethylation of the same histones results in a heterochromatin conformation and transcriptional repression [10]. In this way, the covalent modification of histone tails by acetylation and methylation dynamically shapes the chromatin landscape to regulate gene transcription. The ability of these modifications to be stably inherited by daughter cells after cell division contributes to cellular identity and thus immunological memory.

In memory T cells, histone modifications epigenetically mark genes and prime them for rapid reactivation following exposure to specific antigens (see Table 1). For example, several studies have shown that H3 acetylation at the *IFNG* promoter is maintained from naïve T cells into CD8^+^ memory T cells following viral activation [11,12]. Similarly, chromatin immunoprecipitation (ChIP) analysis showed that active H3K9 acetylation is present at both active and “poised” genes in memory cells [13]. Similarly, CD4^+^ resting memory cells demonstrated increased H3 acetylation at appropriate *IFNG*, *IL-4*, and *IL13* loci upon activation [14,15]. Furthermore, an absence of histone acetylation at the *IFNG* locus in CD8^+^ cells resulted in a loss of memory T cell function, which was restored following hyperacetylation by the histone deacetylase (HDAC) enzyme [11,12].

**Table 1:** **Summary of epigenetic mechanisms and their role in memory cell development**

**Mechanism**	**Cell type/species**	**Role**	**Ref**
**PTMs**			
Acetylation	Memory CD4^+^/CD8^+^ T cells	▪ Marks memory responsive genes for rapid reactivation	[15]
		▪ Form stable marks of transcriptional activation that are retained in memory T cells	[11-14]
Methylation	Memory CD4^+^/CD8^+^ T cells	▪ Loss of repressive histone methylation marks at active genes are preserved in memory T cells to facilitate faster transcription of target genes	[16,17]
**Histone variants**			
H3.3	mES cells	▪ Marks gene enhancers for rapid reactivation	[37,38]
		▪ Primes genes for transcription by destabilising nucleosome structure to facilitate recruitment of transcription factors	
	Xenopus	▪ Required for transcriptional memory following somatic cell transfer	[47]
	pre-B cells	▪ Forms stable marks of transcriptional activity that persist through cell division	[49]
H2A.Z	Yeast/CD4^+^ T cells	▪ Destabilises chromatin structure to facilitate recruitment of transcription machinery	[29-31]
	Yeast	▪ Regulates the localisation of recently repressed genes to the nuclear periphery to facilitate transcriptional memory	[48]
H2A.Lap1	Mouse testis cells	▪ Selectively recruited at the TSS of active genes to destabilise nucleosomal structure and facilitate transcriptional reactivation	[51,52]
**Transcription factors**			
STAT3	Memory CD8^+^ T cells	▪ Regulates the expression of pro-memory transcription factors essential for generation of memory T cells	[65,68]
Tcf1	Memory CD8^+^ T cells	▪ Regulates memory T cell formation and immune responses through the induction of Eomesodermines and regulation of pro-memory transcription factors	[67,68]
FOXO1	Memory CD8^+^ T cells	▪ Translocates into the cell nucleus to regulate the transcription of numerous pro-memory transcription factors	[69-72]
NFAT	Memory CD4^+^ T cells	▪ Regulates transcription of genes that are critical for memory T cell development	[64]
NF-κB	Memory T cells	▪ Regulates transcription of genes that are critical for memory T cell development	[65]
RBPJ	Murine Carcinoma F9 cells	▪ Regulates chromatin domains and long-range chromatin interactions to maintain gene expression programs in transcriptional memory	[85]
**Kinases**			
PKC-θ	T cells	▪ Key regulator of IL-2 expression (a critical cytokine for memory T cell development)	[58]
mTOR kinase	CD8^+^ T cell	▪ Regulates memory CD8 T-cell differentiation through the transcription factors T-bet and Eomesodermin.	[86,69]
**Chromatin remodellers/RNA Pol II machinery/ncRNA/NPC**			
SWI/SNF	Yeast	▪ Prevents ISWI-based enzymes from erasing transcriptional memory	[5]
Nuclear Pore Proteins (Nup100)	Yeast	▪ Nup100 interacts with the promoter for yeast gene INO1 to regulate transcriptional memory.	[82]
piRNAs	C.Elegans	▪ Regulate a multigenerational epigenetic inheritance mechanism.	[87]

ncRNA = non coding RNA; NPC = Nuclear Pore Complex.

As well as acetylation, transcriptional preservation in memory T cells is also associated with histone methylation. Genome-wide analysis of histone methylation in CD4^+^ and CD8^+^ memory T cells has shown a general correlation between the distribution of histone methylation (specifically, H3K4me3 and H3K27me3) and gene expression [16]. Following primary infection of CD8^+^ memory T cells, effector genes (such as *IFNG* and *GZBM*) are transcriptionally up-regulated whilst losing repressive H3K27 epigenetic marks on these genes; these chromatin changes are preserved in subsequent memory T cell generations [16]. Furthermore, histone methylation regulates Th_2_ cell-associated cytokine production in mice lacking the histone methyltransferase HRX (encoded by *Mll*). *Mll*-deficient mice have reduced H3K4 methylation at *Gata3* and *Il-4* loci accompanied by decreased gene expression of both *Gata3* and *Il-4* in CD4^+^ memory T cells [17]. These studies demonstrate that epigenetic signatures in activated effectors can remain present in resting memory T cells, thus showing that histone modifications contribute to memory T cell function.

Genomic analyses of histone PTMs have shown that the epigenetic signatures associated with active and repressed marks are not mutually exclusive. Combinations of PTMs are utilised at different stages of cellular differentiation to regulate distinct transcriptional programs. For example, genome-wide mapping of H3K4me3 (associated with transcriptionally active genes) and H3K27me3 (associated with transcriptionally silent genes) in embryonic stem cells (ESCs) has shown that both marks can reside at the same genomic location; these are known as “bivalent” loci [18,19]. Furthermore, genes that become transcriptionally active upon differentiation maintain H3K4me3 and lose H3K27me3, whilst those that are not transcriptionally active maintain H3K27me3 and lose H3K4me3. These data suggest that bivalent loci provide a regulatory mechanism by which transcription can either be rapidly activated or repressed depending on the differentiation pathway initiated. Bivalent chromatin has also been observed at several genomic loci in memory T cells [16,20]. In resting CD8^+^ memory T cells, *KIAA1804* (mixed lineage kinase 4, a gene involved in Toll-like receptor 4 signalling) has a bivalent chromatin state [20] that is more open and transcriptionally active upon CD8^+^ memory T cell activation [16]. Furthermore, upon differentiation into effector and memory CD8^+^ T cells, the majority of gene loci associated with transcription, replication and cellular differentiation, lose repressive H3K27me3 whilst retaining H3K4me3 [21]. Histone modifications appear to play a key role in forming a blueprint for the acquisition and maintenance of cellular gene expression profiles. Overall, these different epigenetic states (active, bivalent, poised, or repressed) provide a means to regulate gene expression and facilitate the function of memory T cells.

Collectively, these studies suggest that histone PTMs act to epigenetically “poise” genes for polymerase accessibility and transcriptional activity in memory cells, and in doing so provide the molecular basis for the rapid and enhanced effector function necessary for memory T cell responses. It will also be important to investigate the enzymes that mediate these PTM changes (the readers and writers of this code) in the future, to determine how these enzymes regulate transcriptional memory.

### Chromatin structure and the contribution of histone variants to transcriptional memory

In eukaryotic cells, genomic DNA is organised into a highly compacted structure known as chromatin. Chromatin plays a key role in genomic regulation, not least in transcription [22]. The basic repeating unit of chromatin is the nucleosome, consisting of DNA wrapped around a core of histone proteins. Each nucleosome core contains an octameric complex of histone proteins (comprising two molecules each of histones H2A, H2B, H3 and H4, or variants of these core histones) around which 147 base pairs of DNA are wound 1.65 turns [23]. Adjacent nucleosomes are then connected by short segments of “linker” DNA to form long arrays, which in turn undergo several inter-nucleosomal interactions to contribute to higher-order chromatin compaction. Consequently, the highly compacted structure of chromatin (known as heterochromatin) can preclude access of transcriptional proteins, such as RNA polymerase II (Pol II) and DNA-binding transcription factors, to target genes [24]. Therefore, heterochromatin is typically associated with transcriptionally silent or repressed genes [22]. To overcome this obstacle, chromatin must be subjected to nucleosomal reorganisation in order to create ‘open’ accessible regions of DNA (called euchromatin), which enable binding of transcriptional machinery and facilitate active gene transcription [24]. The dynamic interplay between heterochromatin and euchromatin formation is mediated in response to distinct environmental stimuli (e.g., exposure to a specific antigen), to expose or hide target DNA and ultimately regulate gene expression [24]. However, the epigenetic processes that contribute to chromatin reorganisation are still poorly understood.

Currently, three main mechanisms of chromatin remodelling have been characterised, including PTM of histones (discussed in previous section), ATP-dependent chromatin remodelling complexes, and replacement of canonical histones with histone variants; known as “histone variant exchange” [25]. In contrast to replication-dependent canonical histones, which are only deposited during replication, histone variants are expressed and incorporated throughout the cell cycle to directly replace existing core histones or replace histones that have been previously evicted [25]. Variant histone deposition can alter the chromatin landscape by directly altering nucleosome stability [26], disrupting higher-order chromatin structure [27] and/or indirectly by carrying specific PTMs onto the target DNA [28]. Together, the structural differences of variant histones and their associated modifications destabilise interactions with adjacent nucleosomes. Unstable nucleosomes are then easily removed or displaced by regulatory proteins to produce open regions of chromatin and facilitate active transcription of target genes [29].

Whilst many histone variants exist, most research has focused on the variants H2A.Z and H3.3. The histone variant H2A.Z is primarily associated with transcriptional activation. For example, recent studies have shown that H2A.Z is enriched at the inactive promoters of inducible genes, and is subsequently depleted upon transcriptional activation [10,29,30]. Similarly, defined nucleosome-free regions present at the promoters of inducible genes are flanked by two well-positioned H2A.Z-containing nucleosomes, suggesting that the H2A.Z variant helps to poise genes for rapid transcriptional activation [30,31]. Interestingly, whilst histone H2A.Z has primarily been associated with active transcription, few studies have also demonstrated a negative role for H2A.Z in transcription regulation [32,33]. In contrast to H2A.Z, the histone variant H3.3 is commonly associated with transcriptionally active chromatin and is largely deposited at both transcribed regions of DNA and gene promoters [34,35]. Consistently, H3.3 is often enriched in PTMs associated with active chromatin, such as acetylation [28,36]. In addition, recent studies have shown that H3.3 is extensively enriched at enhancer regions, and that H3.3 primes enhancers for active transcription [27,37]. For example, deposition of H3.3 at retinoid acid-regulated gene enhancers in mouse embryonic stem (mES) cells was shown to facilitate binding of the RAR/RXR transcription factor, by impairing higher-order chromatin folding [38]. Furthermore, higher levels of histone H3.3 were observed at the enhancers of poised genes that concomitantly displayed lower levels of active enhancer markers (such as p300; a co-activator of transcription), which suggests that H3.3 may act as an epigenetic marker for poised enhancers [38]. Interestingly, recent work has also found that the H3.3 variant is deposited at sites of transcriptionally inactive chromatin, such telomeric and pericentric heterochromatin in mES cells [35,39,40]. In order to elucidate the role of these histone variants in transcriptional regulation, it is critical to understand where they occur within the nucleosomal template (e.g. promoters or distal regions).

Differences between the amino acid sequences of histone variants and their canonical histones largely determine the dynamics of chromatin structure. The highly unstable histone variant H2A.Z shares ~60% structural similarity with its canonical counterpart H2A. These structural differences are significant enough to generate nucleosomal instability and impair chromatin folding [25]. Specifically, the nucleosomal instability may be attributable to structural difference between histone H2A.Z and H2A in the loop-1 dimerization region [41]. In addition, H2A.Z histone modifications, such as N-terminal acetylation [42] or C-terminal ubiquitylation [43], may also function to regulate nucleosome destabilisation. Unlike H2A.Z, histone H3.3 differs from the canonical histone H3 by only four amino acid residues. However, none of these appear to affect nucleosome stability [44]. Furthermore, loss of histone acetylation [26] and removal of the H3.3 N-terminal tail do not appear to influence inter-nucleosomal interactions or regulate destabilisation [45], suggesting that H3.3-associated PTMs are not involved in chromatin reorganisation. Whilst the effect of histone H3.3 structural properties on chromatin structure is unclear, H3.3 has been shown to greatly impair higher-order chromatin structure [27]. Furthermore, H3.3 has also been found to counteract binding of linker histone H1 in *Drosophila* [46]. It has also been proposed that H3.3-associated histone chaperones and chromatin remodellers may also contribute to histone instability [44]. Interestingly, the heterotypic histone variant H2A.Z/H3.3 has been found to be the most unstable histone variant [34].

Both H2A.Z and H3.3 histone variants have recently emerged as novel regulators of transcriptional memory (Table 1). In particular, these histone variants have been implicated in the regulation of transcriptional memory in lower eukaryotes. For example, pioneering studies in Xenopus revealed that H3.3 and its associated PTM K4me are essential for transcriptional memory of active genes after somatic cell transfer into enucleated eggs [47]. Similarly, H2A.Z is required for the activation of recently repressed genes in yeast, and localisation of these genes at nuclear periphery for rapid reactivation [48]. Furthermore, H3.3 has been shown to form stable marks with H3 acetylation and H3K4me that persist through metaphase chromosomes in pre-B cells [49]. Together, these studies show that histone variants play key roles in the regulation of transcriptional memory. Interestingly, H2A.Z has also been implicated in the formation of cognitive memory; whereupon H2A.Z depletion in mice increases the transcription of specific memory-promoting genes following fear conditioning [50].

Whilst the histone variants H2A.Z and H3.3 have been implicated in transcriptional memory, the contribution of these histone variants to memory T cell development is yet to be defined. However, it is likely that histone variants, together with their associated PTMs, are incorporated into the nucleosomes of inducible genes following activation and mark them for future transcription. Furthermore, it is widely postulated that these stable marks of transcriptional history are retained throughout cell divisions, to facilitate rapid binding of regulatory proteins and faster transcription of immune-responsive genes. Consistently, depletion of the H2A.Z histone variant has been shown to occur concomitantly with the deposition of H3.3 at the promoters of inducible genes in CD4^+^ T cells, suggesting that H2A.Z poises these genes for transcriptional activation [31]. Interestingly, active genes have recently been shown to exhibit selectivity of histone variants at the transcriptional start site (TSS); revealing another layer of complexity in the regulation of transcription [51,52]. In testis cells derived from mice, the histone variant H2A.Lap1 (the mouse homolog of human H2A.Bdb) is selectively enriched at the TSS of active genes, suggesting that specific mechanisms exist to determine the recruitment of variant histones to the nucleosomal template [51]. Therefore, to completely understand the contribution of histone variants to memory T cell development, it will be important to identify histone variant-specific chaperones in future studies. Interestingly, a few histone variant-specific chaperones and chromatin remodelling proteins have recently been identified, which target specific variants for recruitment and facilitate subsequent nucleosomal exchange [53-55].

The dynamics of histone variant exchange in transcriptional memory are poorly defined, particularly in T cells. However, given that the histone variants H2A.Z and H3.3 are cell-cycle independent, highly unstable, and carry active PTMs, it is likely that they play a key role in the regulation of T cell memory. Furthermore, the presence of these unstable histone variants at inactivate regulatory regions of inducible genes suggests that they poise genes for rapid reactivation. Together these characteristics of histone variants strongly indicate a memory cell phenotype. Therefore, future studies should aim to identify specific regulators of H2A.Z and H3.3 (utilising methods such as ChIP), to further understand the contribution of these histone variants to transcriptional memory in T cells.

### Signalling kinases as novel epigenetic regulators of transcriptional memory

Stimulation of memory T cells results in the activation of many protein kinase signalling pathways such as protein kinase C (PKC). PKC is an evolutionarily conserved signalling kinase that catalyses the phosphorylation of numerous downstream signalling targets (e.g., transcription factors), and is a key regulator of gene expression in T cells [56]. Many different PKC isozymes exist which regulate specific signalling pathways and generate distinct outcomes of gene expression. Of these isozymes, PKC-theta (PKC-θ) is the most abundantly expressed in T cells [57].

PKC-θ is an important regulator of many key biochemical events in T cells, such as activation and proliferation, effector functions, and survival [56]. For example, PKC-θ knockdown inhibits expression of IL-2 (an important T cell activation cytokine), preventing subsequent T cell proliferation and differentiation into effector and memory populations (Table 1) [58]. Similarly, PKC-θ knockdown significantly decreases transcription of cytokine genes in virus-activated CD4^+^ and CD8^+^ T cells [59]. Furthermore, PKC-θ has been shown to be a critical signalling molecule for T cell survival. Enhanced CD8^+^ T cell apoptosis and reduced expression of survival proteins is observed in PKC-θ-deficient mice following viral infection [60]. Given the important role of PKC-θ in T cells, it is likely that this kinase also regulates transcription factors involved in establishing and maintaining transcriptional memory in memory T cells.

Although PKCs were initially thought to function exclusively in the cytoplasm, recent studies have shown that PKC-θ and PKC-β form a novel class of nuclear epigenetic enzymes in mammalian cells [61,62]. Pioneering studies in yeast demonstrated that signalling kinases are able to translocate to the nucleus and directly associate with chromatin to regulate gene expression [63,64]. For example, nuclear-anchored PKC-θ forms an active nuclear signalling complex by interacting with Pol II, the histone kinase MSK-1, the adapter molecule 14-3-3 zeta, and the histone methylation eraser enzyme lysine-specific demethylase 1 (LSD1) on immune-responsive gene promoters in activated human T lymphocytes [61]. Similarly, PKC-β1 also has a nuclear role, with chromatin-bound PKC-β1 phosphorylating H3T6 to sequentially inhibit the functional activities of LSD1 on H3K4 [62]. Nuclear PKC-θ provides more efficient regulation of immune responsive genes in activated T cells. The dual role of PKC-θ represents an additional layer of gene regulation, and, in this way, PKC may potentially regulate T cell memory from a signalling and/or chromatin-associated perspective.

### Transcription factors and their role in transcriptional memory

Whilst the exact molecular mechanisms that underpin transcriptional memory are slowly becoming clear, the transcription factors that regulate transcriptional memory remain poorly defined. However, several transcription factors have recently been identified to play a key role in memory T cell development (Table 1). For example, following activation Stat3-deficient T cells undergo terminal differentiation and fail to form self-renewing T_CM,_ suggesting that this signalling pathway is important for memory T cell generation [65]. Consistently, patients with autosomal-dominant hyper-IgE syndrome (a disease often caused by dominant-negative mutations in *STAT3*) form decreased numbers of T_CM_ cells and display defective immune responses against viral infections [68]. In addition to Stat3, deletion of the transcription factor *Tcf1* has been shown to promote CD8^+^ T cell differentiation into short-lived effector T cells and impair the maintenance memory precursors, resulting in decreased T_CM_ cells and impaired immune responses to pathogen re-challenge [67,68]. Recent studies have also identified the forkhead box O1 (Foxo1) transcription factor as a key regulator of CD8^+^ T cell effector differentiation via AKT/mTOR signalling. Loss of *Foxo1* is associated with reduced transcription of pro-memory transcription factors (such as Eomesodermins, Bcl-6, T-bet), as well as down-regulation of the lymph node-homing molecule Klf-2 (critical for T_CM_ cell function) [69-72]. Similarly, the transcription factors NFAT [73] and NK-κB [74] have also been implicated in T cell memory, displaying enhanced activation in memory CD4^+^ T cells compared to naïve CD4^+^ T cells. Interestingly, whilst MAP kinases ERK, JNK and p38 [75,76] are phosphorylated more efficiently and increased levels of upstream regulator ZAP-70 [77] are observed in memory T cells, these observations are not linked to the epigenomic setting up of transcriptional memory. Whilst recent studies have identified and characterised novel transcription factors and signalling pathways involved in T cell transcriptional memory, our understanding of these transcription factors in the regulation of T cell memory is still in its infancy.

### Yeast as a model system for transcriptional memory

Although T cells have proven to be an excellent model for transcriptional memory, yeast cells also represent an elegant system for studying transcriptional memory. Epigenomic research in yeast has greatly improved our understanding of the molecular mechanisms that regulate this process (Table 1). Whilst studies on transcriptional memory have primarily focused on histone PTMs and histone variants, a variety of other molecular factors have recently been associated with transcriptional memory in yeast. For example, transcriptional memory in yeast has been associated with the formation of gene loops (termed ‘memory gene loops’; a looped gene conformation formed by the interaction of the gene promoter and 3’ end), which facilitates the binding of Pol II upon transcriptional activation [78]. Inducible genes are able to maintain these memory gene loops during periods of short-term repression, therefore, these genes can be more rapidly and robustly transcribed following reactivation [78]. Interestingly, the feature of gene looping has also been observed at human genes, such as *BRCA1* [79]. However, the function of gene looping in human cells has yet to be defined.

In addition, chromatin remodelling enzymes have also been shown to play a key role in the regulation of transcriptional memory in yeast. Deletion of the *SWI2* gene in yeast, which encodes the SWI/SNF ATP-dependent remodelling enzyme, abolished *GAL1* transcriptional memory following secondary exposure to galactose [5,80]. Similarly, the regulation of *GAL1* transcriptional memory has also been associated with the cytoplasmic factor, Gal3, which is produced upon initial galactose induction [81].

Furthermore, transcriptional memory has also been associated with the localisation of at inducible genes at the nuclear periphery. Many inducible genes in yeast, such as *GAL1* and *INO1,* relocate to the nuclear periphery and associate with the nuclear pore complex upon activation [82]. The nuclear pore protein interacts with promoters of recently repressed genes to promote chromatin reorganisation and binding of Pol II, therefore, poising these genes for rapid reactivation [82-84]. Similarly, localisation of *IFNG* at the nucleoplasm is required for transcriptional memory in human cells; depletion of Nup100 resulted in the loss of *IFNG* transcriptional memory in HeLa cells [84].

Together, these observations lead us to ask whether these mechanisms are conserved in memory T cells, and other eukaryotes that display transcriptional memory. Therefore, future studies should aim to examine specific mechanisms employed for transcriptional memory in yeast, such as gene looping and nuclear localisation, in the context of memory T cells. It may be that memory T cells preferentially utilise certain mechanisms over others, or that they do not utilise these mechanisms at all. Transcriptional memory in T cells requires greater comprehensive characterisation, and yeast presents an elegant solution for understanding the underlying molecular mechanisms involved in memory cell development.

### Conclusion: a multi-layered model of transcriptional memory

**Figure 1:**
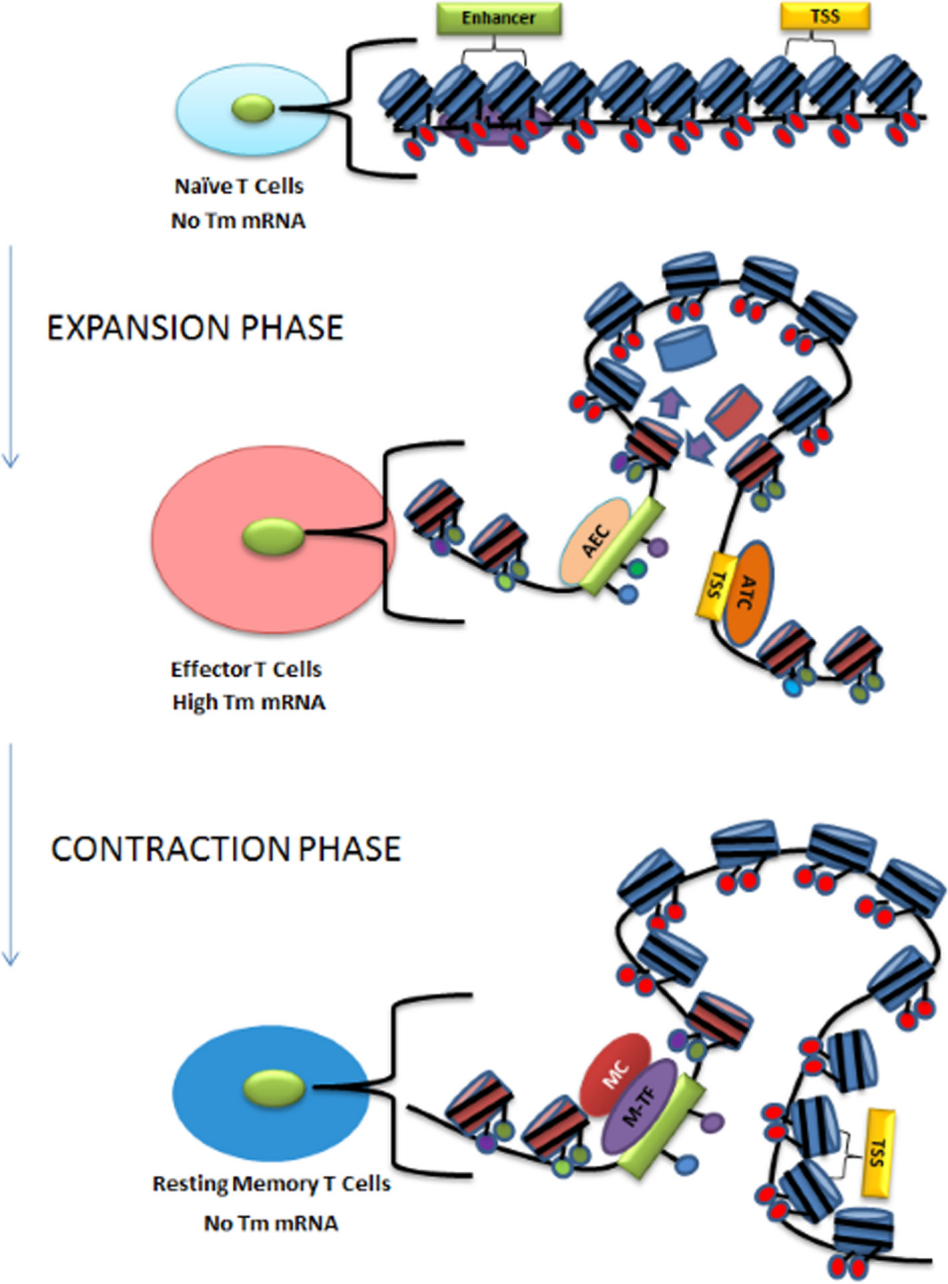
Model depicting the existence of T cells in distinct chromatin states in transcriptional memory responsive genes that allow for rapid and robust gene induction in memory T cells. In response to viral infection, naïve T-cells rapidly expand into effector T-cells and subsequently contract to produce a small population of resting, long-lived memory T cells. These memory cells have the ability to express genes more rapidly and robustly than effector T-cells; a feature known as transcriptional memory (Tm). In this multi-layered model of transcriptional memory, we envisage a scenario whereby multiple epigenetic mechanisms, such as PTMs, histone variants, transcription factors, gene looping, localisation of genes within the nucleus, and the regulatory elements themselves, collectively contribute to the transcriptional memory response in T cells. In the above Figure, H3/H2A nucleosomes are represented by *blue cylinders* and repressive PTMs are *red balls*; H2AZ/H3.3 nucleosomes are represented by *red cylinders* and active PTMs are signified by *green, purple* or *blue coloured balls*. The active transcription complex (ATC) is signified by an *orange oval* and the active enhancer complex (AEC) by a *tan oval*, each representing transcription factors (TF), PKC-θ, LSD1, Pol II and other unidentified members which are bound to the promoter region/TSS (TSS signified by a *yellow box*) or enhancer region (signified by a *green box*). The *purple oval* represents the memory transcription factors (M-TF). The memory complex (MC) is signified by a *red oval* representing unidentified members and Pol II. The above Figure also depicts the formation of a chromatin loop following activation, which allows the enhancer to interact with promoter. The chromatin loop relocates to the nuclear periphery upon activation, were it remains in resting memory T cells.

Over the past decade, research exploring the regulation of memory T cell development has primarily focused on histone PTMs, eraser/writer enzymes, signalling enzymes, and nucleosome configuration and structure. However, recent studies in yeast have identified several novel mechanisms of transcriptional memory regulation, such as histone variant exchange, the formation of memory gene loops, and localisation of memory-responsive genes to the nuclear periphery. Based on these observations, we propose a multi-layered model of transcriptional memory, in which numerous epigenetic mechanisms work together to establish and maintain transcriptional memory in memory T cells (see Figure 1 Multi-layered model of transcriptional memory). In this model, inducible genes are transcriptionally silent in naïve T cells, existing in a heterochromatin state decorated with stable canonical histones (such as H2A/H3) which carry repressive PTM signatures. Upon antigen-mediated activation, specific epigenetic proteins, such as memory-specific transcription factors, signalling kinases, and chromatin chaperone/remodelling proteins, work together to establish an open euchromatin conformation by adding active histone PTMs and incorporating unstable histone variants (e.g., H2A.Z/H3.3) at regulatory regions (i.e., promoters and enhancers), as well as throughout the target gene. In addition, we hypothesise that T cell activation also induces the formation of memory gene loops, localisation of inducible genes to the nuclear periphery, and the recruitment of an active transcription complex to the TSS. Together, these epigenetic mechanisms form an active signature of transcriptional memory that persists into memory T cell populations, and functions to epigenetically ‘poise’ previously activated genes for more rapid and robust transcription upon reactivation.

Together, these mechanisms provide multiple layers of regulation that ultimately dictate gene behaviour and contribute to transcriptional memory. Transcriptional memory is an evolutionarily conserved process; therefore, identifying the mechanisms underlying transcriptional memory in other memory processes, such as cognitive memory, can help further understand this process in memory T cells. Understanding the molecular mechanisms that underpin the rapid recall responses of memory T cells is a critical component for the development of safer and more effective vaccines.

## Abbreviations

T_CM_ cell: Central memory T cell
T_EM_ cell: Effector memory T cell
IL-2: Interleukin-2
TNF: Tumour necrosis factor
IFNG: Interferon-γ
PTM: Post-translational modification
ChIP: Chromatin immunoprecipitation
HDAC: Histone deacetylase
ESC: Embryonic stem cell
Pol II: RNA polymerase II
mES cells: Mouse embryonic stem cells
TSS: Transcriptional start site
PKC: Protein kinase C
PKC-θ: PKC-theta
LSD1: Lysine-specific demethylase 1
FOXO1: Forkhead box O1
